# Distinct association between cerebral arterial pulsatility and subtypes of cerebral small vessel disease

**DOI:** 10.1371/journal.pone.0236049

**Published:** 2020-07-16

**Authors:** Ki-Woong Nam, Hyung-Min Kwon, Yong-Seok Lee

**Affiliations:** 1 Department of Neurology, Seoul Metropolitan Government-Seoul National University Boramae Medical Center, Seoul, Korea; 2 Seoul National University College of Medicine, Seoul, Korea; Ehime University Graduate School of Medicine, JAPAN

## Abstract

**Background:**

Increased arterial resistance is a potential pathological mechanism of cerebral small vessel disease (cSVD).

**Aim:**

In this study, we aimed to investigate the association between pulsatility index (PI) representing cerebral arterial resistance and subtypes of cSVD in patients with lacunar stroke.

**Methods:**

We included consecutive lacunar stroke patients between 2010 and 2013. White matter hyperintensity (WMH) volume was rated using semi-automated quantitative methods. Additionally, the presence of old lacunar infarct (OLI), cerebral microbleed (CMB), or enlarged perivascular space (EPVS) was also evaluated. The relationship between PI, measured in each middle cerebral artery, and the subtype/burden of cSVD was analyzed in the relevant hemisphere.

**Results:**

A total of 206 lacunar patients were included and 412 hemispheres were analyzed (mean age: 64 years, male: 68.4%). In multivariable analysis, PI was positively associated with the WMH volume [beta = 1.372, 95% confidence interval (CI) = 0.624 to 2.120, *P* < 0.001] after adjusting for confounders. PI was also related to the presence of OLI (adjusted odds ratio = 11.37, 95% CI = 2.55–48.56, *P* = 0.001); however, this relationship was not significant in CMB or EPVS. Regarding the cSVD burden, PI increased according to the WMH tertiles (*P* for trend < 0.001), the burden of OLI (*P* for trend < 0.001), and EPVS tertiles (*P* for trend < 0.001), showing a quantitative relationship.

**Conclusions:**

Ipsilateral PI is closely associated with cSVD in patients with lacunar stroke. Furthermore, this association is different between subtypes of cSVD, which is suggestive of underlying pathophysiological differences.

## Introduction

Cerebral small vessel disease (cSVD) is a subclinical pathology with various subtypes [e.g., white matter hyperintensity (WMH), old lacunar infarct (OLI), cerebral microbleed (CMB), and enlarged perivascular space (EPVS)] [1, 2]. These subtypes appear to have very different pathologies, but they are often found together and were correlated with each other according to previous autopsy studies [1, 3, 4]. Since cSVD is responsible for dementia and stroke [1, 5], efforts have been made to identify common pathologic mechanisms among cSVD subtypes [3, 4]. Several possible mechanisms have been suggested, and increased arterial resistance is one of them [6, 7].

Advancing age or prolonged exposure to hypertension causes systemic vascular remodeling [8–11]. As elastic components decrease, muscle cells proliferate, and extracellular matrix accumulates, arteries become stiffer increasing systemic arterial resistance [8, 10, 11]. Stiff arteries will not reduce pulse energy by Windkessel effect, and as a result, systemic pulse energy will be delivered to perforating arterioles as it is [1, 8, 9, 11–13]. This phenomenon has been actually demonstrated by the results of various experiments that examine the change of waveform in stiff artery, and mathematical models that predict it [14–16]. Also, arterial stiffness has been mainly measured by the carotid-femoral or brachial-ankle pulse wave velocity, and many previous studies have shown close relationships with cSVD [6, 17]. This would be another indirect evidence that the pulse energy is directly transmitted to perforating arterioles in stiff arteries.

Transcranial Doppler sonography (TCD) has also employed as a non-invasive tool to measure arterial resistance. When the vascular resistance increases at the distal point than the position (i.e., downstream resistance) we estimated by TCD, a peaked waveform is observed, a slight decrease in peak systolic velocity and a prominent decrease in end diastolic velocity appear [10, 14, 18]. Consequently, as suggested by Gosling, the pulsatility index (PI) using the ratio of these velocities reflects the downstream vascular resistance distal to the examined artery [19], thus; making it possible to measure the pathologies of intracranial small arteries/arterioles (i.e., cSVD) in the proximal large artery [20, 21]. Using PI values measured by TCD or magnetic resonance imaging (MRI), many studies have evaluated the relationship between PI and cSVD [10, 22, 23]. The close associations of PI with WMH or OLI have been confirmed in a few studies, but no studies have been conducted across PI and cSVD subtypes [8, 20, 21, 24–26].

In this study, we evaluated the relationship between PI and cSVD in patients with lacunar stroke. We also assessed whether increased arterial resistance is a common pathological mechanism involved in all cSVD subtypes or if it behaves differently depending on the subtype.

## Material and methods

### Patients and participants

From a consecutive stroke registry at a large stroke center in Korea (Seoul Metropolitan Government-Seoul National University Boramae Medical Center) between January 2010 and December 2013, we included patients with first-ever ischemic stroke within 7 days of symptom onset (n = 959). Among them, we sorted out lacunar stroke, based on the Trial of ORG 10172 in Acute Stroke Treatment classification (n = 302) [27]. Patients with intracranial atherosclerosis, extracranial atherosclerosis, and high-risk cardioembolic sources were excluded from the lacunar stroke screening process [27]. As additional exclusion criteria, patients who 1) had no brain MRI data (n = 12), 2) were <18 years of age (n = 9), and 3) had incomplete TCD examinations, including poor temporal window (n = 75) were excluded. Finally, a total of 206 patients were considered for final analyses.

This study was approved by the institutional review board at Seoul Metropolitan Government-Seoul National University Boramae Medical Center (number: 10-2018-64). This study was designed as a retrospective study in which medical records were only reviewed. Thus, informed consent was not needed and even unattainable. Understanding this problem, the IRB of Seoul Metropolitan Government-Seoul National University Boramae Medical Center approved this study, despite not having informed consent.

### Clinical assessment

All patients who are diagnosed as ischemic stroke are principally admitted and undergo broad etiological evaluations including brain MRI, magnetic resonance angiography, echocardiography, electrocardiogram, and laboratory examinations. We also assessed the demographic, clinical, and vascular risk factors, including age, sex, hypertension, diabetes, hyperlipidemia, current smoking, use of antihypertensives, use of lipid-lower agents, and initial stroke severity [28]. Initial stroke severity was assessed based on the National Institutes of Health Stroke Scale (NIHSS) score by well-trained neurologists at the time of admission.

### Radiological assessment

In this study, we conducted brain MRI and magnetic resonance angiography in all participants within 24 hours of admission using a 3.0-T MR scanner (Achieva 3.0T; Philips, Eindohoven, the Netherlands). To evaluate each subtype of cSVD, we measured WMH volume using a computer-assisted semi-automated method with Medical Imaging Processing, Analysis, and Visualization (MIPAV, version 7.3.0, National Institutes of Health; Bethesda, MD) [28]. To investigate the quantitative relationship between PI and cSVD subtypes, we divided our cohort into thirds according to the WMH volume (i.e., WMH tertiles). Old lacunar infarct (OLI) was defined as an asymptomatic well-defined lesion, 3–15 mm in size, with the same signal characteristics as the cerebrospinal fluid in the territorial area of a perforating arteriole [2]. We defined CMBs as focal round lesions smaller than 10 mm with low signal on T2-gradient echo images [2]. The burden of OLI and CMB was classified as absent, single, and multiple according to their numbers. Enlarged perivascular space (EPVS) was defined as a round, oval, or linear lesion smaller than 3 mm with a signal similar to that of cerebrospinal fluid without a surrounding hyperintense rim [2]. Since EPVSs at the basal ganglia level are known to be closely related to other cSVD subtypes, we rated the number of EPVSs at this level [28]. In previous studies, EPVS number was classified into 0 to 10, 11 to 25, and > 25 groups in bilateral hemispheres [28, 29]. However, considering the spatial relationship with PI, all cSVD subtypes and PI values in this study were measured for each hemisphere. There is no reference data on how to divide the burden of EPVS in a single hemisphere. Thus, we decided to divide the cohort into three groups according to the number of EPVSs (i.e., EPVS tertiles). All radiological assessments were rated by two well-trained neurologists (K.W.-N. and H.-M.K.) and disagreements were resolved by discussion with a third rater (Y.-S.L.).

### Transcranial Doppler sonography

**Fig 1:**
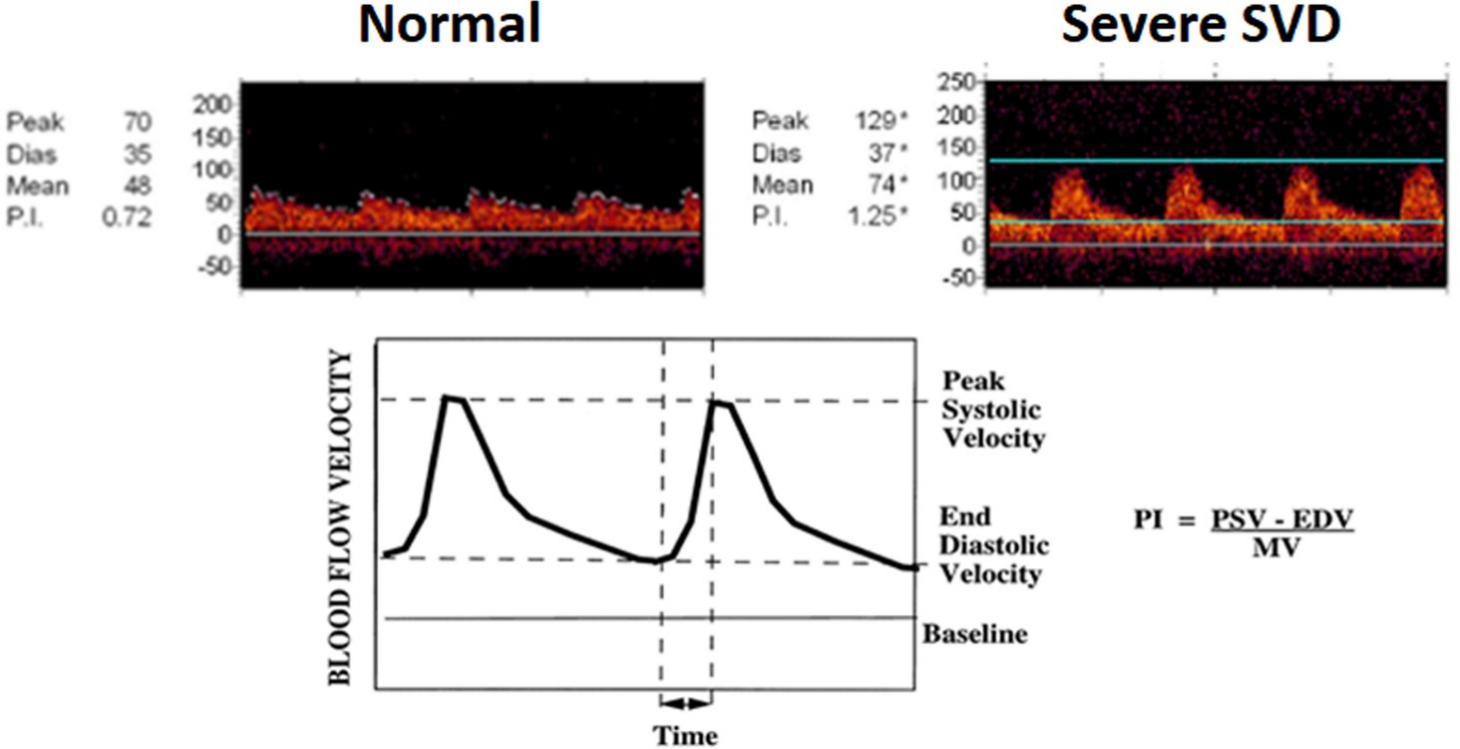
Diagram for calculating Pulsatility Index (PI). A. Normal to mild SVD case: Initially, MCA was identified as identified as flow toward the probe from the temporal windows within an insonation depth of 50–60 mm. By slowly increasing insonation depth to 40 mm, bifurcation of the terminal internal carotid artery is identified. From this point, the entire length of M1 flow is evaluated until the signal is divided into two or three branches (M2 of MCA). Peak systolic velocity, end diastolic velocity, and mean flow velocity were obtained from proximal to distal M1 with 5 mm intervals of insonation depth. B. Severe SVD case: Patients with severe SVD have peaked shape waveforms, slight PSV reduction, and prominent EDV reduction. This results in increased PI value. C. PI was calculated as [PI = (peak systolic velocity–end diastolic velocity)/mean flow velocity].

The TCD evaluations were performed using a TCD monitoring device (Spencer; PMD 150, United States) with two-2MHz probes, fixed in a metal headframe (Spencer, Marc 1500, United States), within 7 days after admission [30]. The protocol was standardized for every patient, and conducted by skilled sonographers [30]. Data including peak systolic velocity, mean flow velocity, and PI were obtained along the M1 segment of the middle cerebral artery (from the temporal windows within an insonation depth of 50–60 mm). To calculate PI, the Gosling’s equation [PI = (peak systolic velocity–end diastolic velocity)/mean velocity] was used (Fig 1) [19]. Both mean flow velocity and PI were measured in each hemisphere, respectively.

### Statistical analysis

As univariate analyses, we used simple linear regression for WMH volume and simple logistic regression for OLI, CMB, and the highest EPVS tertile to determine possible predictors for cSVD. In the case of EPVS, as it is unlikely that 1–2 lesions make a substantial clinical difference, the authors used the highest tertile as the criteria for the outcome. Continuous variables with skewed data were transformed into a square root scale before analysis. Variables with *P* values < 0.05 in the univariate analyses and diffusion-weighted imaging volumes were introduced into the multivariable linear and logistic regression analyses, respectively. The size of EPVSs is smaller than other pathologies such as WMH, OLI, and CMB. Therefore, to avoid masking the relationship with PI by the size of the other accompanying pathologies, we conducted additional multivariable analyses for EPVS. To this aim, we included only patients with mild burden of cSVD as 1) within 1^st^ tertile of WMH volume; 2) absent to single OLI; and 3) absent to single CMB.

To evaluate the quantitative relationships between PI and various cSVD subtypes, we compared PI values among individuals with different burdens of each cSVD subtype (WMH, OLI, CMB, and EPVS). In this analysis, we used the Jonckheere-Terpstra test. Additionally, to understand the mechanisms that connect PI and cSVD, we evaluated the association between the PI and vascular risk factors using simple linear regression analysis. All statistical analyses in this study were performed using SPSS version 21.0 (IBM, SPSS, Chicago, IL, USA). *P* values < 0.05 were considered statistically significant.

## Results

A total of 206 lacunar stroke patients were included and 412 hemispheres were evaluated (Median age: 64 [55–72] years, male sex: 68.4%, initial NIHSS score: 2 [1–4]). The median PI level was 0.89 [0.79–1.02] and the median volume of WMH was 2.03 [0.78–6.24] mL. The prevalence of OLI and CMB was 149 (36.2%) and 109 (26.5%), respectively. The median number of EPVS in each hemisphere was 5 [2–10]. Other baseline characteristics of the cohort are presented in Table 1. The PI in this cohort was related to age, diabetes, current smoking, WMH volume, OLI, and EPVS number (S1 Table).

**Table 1:** Baseline characteristics of the cohort (n = 206).

Clinical findings (in 206 patients)	
Age, y [IQR]	64 [55–72]
Visit time, day [IQR]	2 [1–4]
Sex, male, n (%)	141 (68.4)
Hypertension, n (%)	129 (62.6)
Diabetes, n (%)	61 (29.6)
Hyperlipidemia, n (%)	72 (35.0)
Current smoking, n (%)	84 (40.8)
Use of antihypertensives, n (%)	99 (48.3)
Use of lipid-lowering agents, n (%)	156 (75.7)
Initial NIHSS score, [IQR]	2 [1–4]
Radiological findings (in 412 hemispheres)	
Initial diffusion-weighted imaging volume, mL [IQR]	0.45 [0.18–1.17]
White matter hyperintensity, mL [IQR]	2.03 [0.78–6.24]
Enlarged perivascular space, [IQR]	5 [2–10]
Old lacunar infarcts, n (%)	149 (36.2)
Cerebral microbleeds, n (%)	109 (26.5)
Sonographic findings (in 412 hemispheres)	
Mean flow velocity, cm/s [IQR]	59 [50–69]
Pulsatility index, [IQR]	0.89 [0.79–1.02]

NIHSS = National Institutes of Health Stroke Scale.

In univariate and multivariable linear regression analyses, ipsilateral PI was positively associated with WMH volume [beta = 1.372, 95% confidence interval (CI): 0.624 to 2.120; *P* < 0.001] after adjusting for confounders (Table 2). Age (beta = 0.038, 95% CI: 0.026 to 0.050; *P* < 0.001) and hypertension (beta = 0.334, 95% CI: 0.095 to 0.573; *P* = 0.006) also showed positive correlation with WMH volume, while hyperlipidemia (beta = -0.326, 95% CI: -0.571 to -0.081; *P* = 0.009) and current smoking (beta = -0.292, 95% CI: -0.548 to -0.037; *P* = 0.025) had negative association.

**Table 2:** Simple and multiple linear regression analyses between possible predictors and white matter hyperintensity volume*.

	Univariate analysis		Multivariable analysis	
	Beta (95% CI)	*P* value	Beta (95% CI)	*P* value
Age	0.056 (0.046 to 0.065)	< 0.001	0.038 (0.026 to 0.050)	< 0.001
Sex, male	-0.195 (-0.484 to 0.094)	0.185	…	…
Hypertension	0.418 (0.143 to 0.692)	0.003	0.334 (0.095 to 0.573)	0.006
Diabetes	0.137 (-0.157 to 0.431)	0.360	…	…
Hyperlipidemia	-0.464 (-0.742 to -0.186)	0.001	-0.326 (-0.571 to -0.081)	0.009
Current smoking	-0.722 (-0.986 to -0.458)	< 0.001	-0.292 (-0.548 to -0.037)	0.025
Initial NIHSS*	0.110 (-0.092 to 0.311)	0.286	…	…
Use of antihypertensives	0.469 (0.203 to 0.736)	0.001	…	…
Use of lipid-lowering agents	-0.022 (-0.335 to 0.292)	0.891	…	…
DWI volume*	-0.033 9–0.165 to 0.099)	0.623	-0.018 (-0.131 to 0.095)	0.755
Pulsatility index	2.879 (2.192 to 3.566)	< 0.001	1.372 (0.624 to 2.120)	< 0.001

NIHSS = National Institutes of Health Stroke Scale, DWI = diffusion-weighted imaging

*These variables were transformed into a square root scale.

In univariate logistic regression analyses, both OLI [adjusted odds ratio (aOR) = 11.08, 95% CI: 3.44–35.68; *P* < 0.001] and the highest EPVS tertile (aOR = 17.46, 95% CI: 5.22–58.37; *P* < 0.001) were associated with PI (Table 3). However, after adjusting confounders, the PI only showed close association with OLI [adjusted odds ratio (aOR) = 11.37, 95% CI: 2.55–48.56; *P* = 0.001] in multivariable logistic regression analysis (Table 4). Nevertheless, if we conducted additional analysis including only patients with mild burden of cSVD, PI was also related to the highest EPVS tertile (aOR = 18.09, 95% CI: 1.28–254.96; *P* = 0.032) (S2 Table). PI did not have significant relationship with CMB (Tables 3 and 4).

**Table 3:** Univariate logistic regression analyses between possible predictors and old lacunar infarct, cerebral microbleed, and the 3^rd^ tertile enlarged perivascular space.

	OLI		CMB		3^rd^ tertile EPVS	
	OR (95% CI)	*P* value	OR (95% CI)	*P* value	OR (95% CI)	*P* value
Age	1.02 [1.01–1.04]	0.011	1.03 [1.01–1.05]	0.002	1.07 [1.05–1.09]	< 0.001
Sex, male	1.94 [1.23–3.07]	0.004	0.82 [0.51–1.30]	0.386	0.91 [0.59–1.41]	0.676
Hypertension	2.09 [1.35–3.23]	0.001	1.72 [1.07–2.77]	0.025	2.21 [1.41–3.44]	< 0.001
Diabetes	1.21 [0.79–1.88]	0.384	0.68 [0.41–1.12]	0.126	0.89 [0.57–1.39]	0.595
Hyperlipidemia	0.68 [0.44–1.05]	0.083	0.71 [0.44–1.14]	0.154	0.68 [0.44–1.05]	0.084
Current smoking	0.97 [0.64–1.46]	0.874	0.43 [0.26–0.69]	0.001	0.43 [0.28–0.66]	< 0.001
Initial NIHSS*	0.90 [0.67–1.22]	0.497	1.21 [0.87–1.68]	0.265	1.66 [1.21–2.29]	0.002
Use of antihypertensives	1.90 [1.26–2.86]	0.002	2.07 [1.32–3.24]	0.001	1.79 [1.19–2.70]	0.006
Use of lipid-lowering agents	0.90 [0.57–1.44]	0.661	0.65 [0.40–1.07]	0.090	0.78 [0.49–1.25]	0.301
DWI volume*	0.96 [0.78–1.17]	0.667	1.00 [0.80–1.24]	0.980	0.98 [0.80–1.20]	0.862
Pulsatility index	11.08 [3.44–35.68]	< 0.001	1.96 [0.60–6.42]	0.267	17.46 [5.22–58.37]	< 0.001

OLI = old lacunar infarct, CMB = cerebral microbleed, EPVS = enlarged perivascular space, NIHSS = National Institutes of Health Stroke Scale, DWI = diffusion-weighted imaging.

*These variables were transformed into a square root scale.

**Table 4:** Multivariable logistic regression analyses between possible predictors and old lacunar infarct, cerebral microbleed, and the 3^rd^ tertile enlarged perivascular space.

	OLI^†^		CMB^‡^		3^rd^ tertile EPVS^§^	
	aOR (95% CI)	*P* value	aOR (95% CI)	*P* value	aOR (95% CI)	*P* value
Age	1.01 [0.98–1.03]	0.630	1.03 [1.00–1.05]	0.042	1.06 [1.03–1.08]	< 0.001
Sex, male	2.10 [1.31–3.39]	0.002	…	…	…	…
Hypertension	2.13 [1.36–3.35]	0.001	1.61 [0.99–2.62]	0.054	2.26 [1.40–3.66]	0.001
Diabetes	…	…	…	…	…	…
Hyperlipidemia	…	…	…	…	…	…
Current smoking	…	…	0.54 [0.32–0.91]	0.020	0.77 [0.47–1.27]	0.304
Initial NIHSS*	…	…	…	…	1.65 [1.18–2.30]	0.004
Use of antihypertensives	…	…	…	…	…	…
Use of lipid-lowering agents	…	…	…	…	…	…
DWI volume*	0.99 [0.80–1.23]	0.950	0.99 [0.79–1.24]	0.927	0.96 [0.75–1.21]	0.700
Pulsatility index	11.37 [2.55–48.56]	0.001	0.67 [0.16–2.81]	0.581	3.00 [0.70–12.80]	0.138

OLI = old lacunar infarct, CMB = cerebral microbleed, EPVS = enlarged perivascular space, NIHSS = National Institutes of Health Stroke Scale, DWI = diffusion-weighted imaging.

*These variables were transformed into a square root scale.

^†^Adjusted with *P* < 0.05 in the univariate analysis (age, male sex, hypertension, and pulsatility index) and DWI volume.

^‡^Adjusted with *P* < 0.05 in the univariate analysis (age, hypertension, current smoking, and pulsatility index) and DWI volume.

^§^Adjusted with *P* < 0.05 in the univariate analysis (age, hypertension, current smoking, initial NIHSS score, and pulsatility index) and DWI volume.

**Fig 2:**
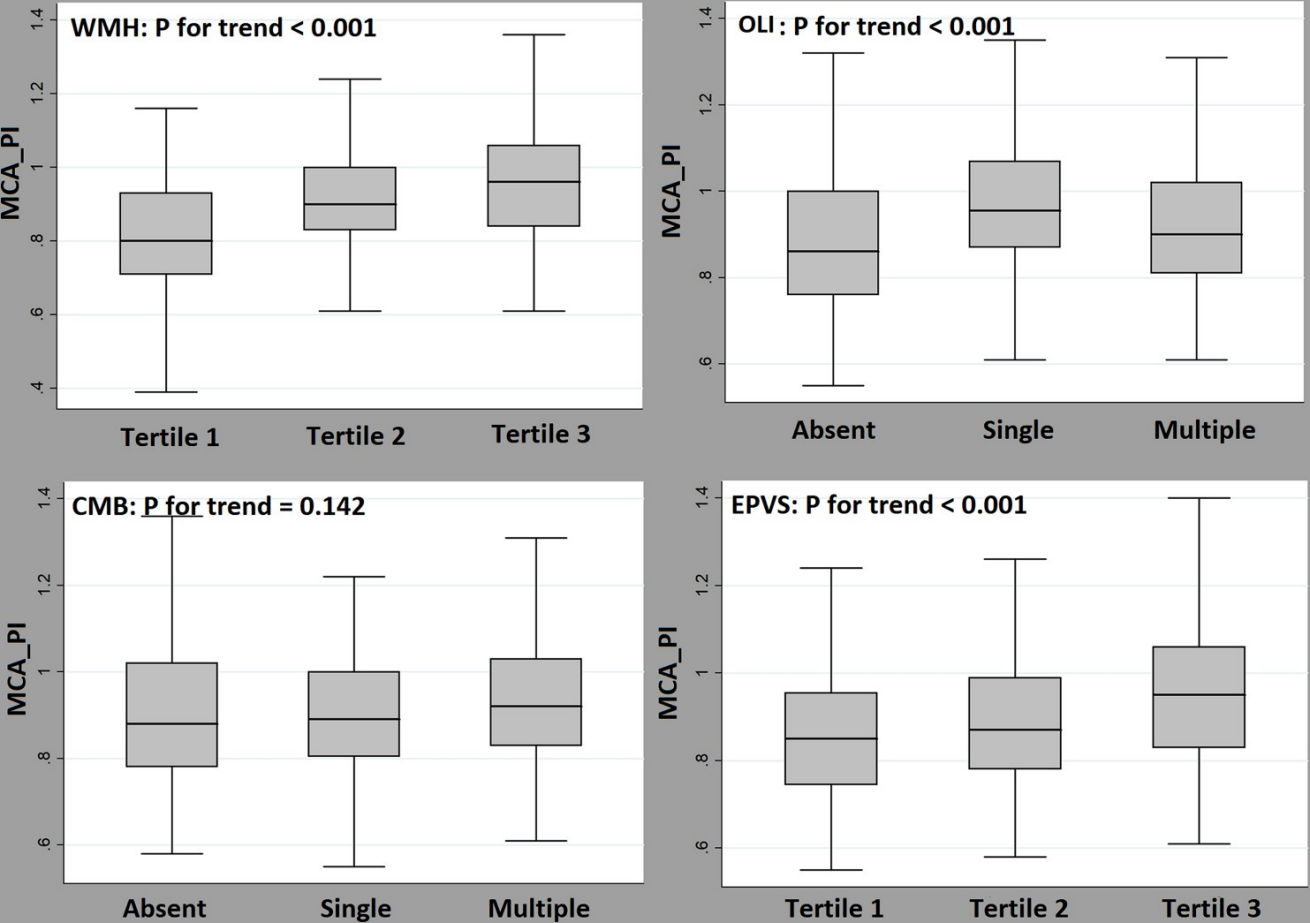
Distribution of mean PI levels according to the burdens of white matter hyperintensity volume, old lacunar infarcts, cerebral microbleeds, and enlarged perivascular spaces. The PI shows positive quantitative associations with WMH volume tertile (*P* for trend < 0.001), number of OLI (*P* for trend < 0.001), and EPVS burden (*P* for trend < 0.001). No association between PI and CMB can be observed (*P* for trend = 0.142).

The evaluation of the relationship between the PI and the burden of the cSVD revealed a positive quantitative correlation between PI level and the WMH volume tertile (*P* for trend < 0.001), number of OLI (*P* for trend < 0.001), and tertile of EPVS number (*P* for trend < 0.001) (Fig 2).

## Discussion

In this study, we found that ipsilateral PI was closely associated with cSVD, especially with WMH and OLI, in patients with lacunar stroke. Since this association with PI differed according to the cSVD subtypes, the pathological mechanism behind the increased arterial resistance may be involved in different ways depending on the cSVD subtypes.

The exact mechanisms explaining this relationship between PI and cSVD are unclear; however, we suggest several hypotheses. First, underlying stiff vessels, expressed in increased PI, may lead to cSVD development. As mentioned earlier, stiff arteries lose their ability of cerebral autoregulation. Prolonged exposure to high and fluctuating pulse energy in the cerebral arterioles can induce damage to the vascular walls, leading to endothelial dysfunction and lipohyalinosis [1, 8, 9, 11, 24]. In addition, the loss of dampening lowers blood pressure below normal in the diastolic phase, which causes the brain to be frequently exposed to chronic hypoxic conditions [8, 13]. Moreover, unstable pulse pressure transmission also inhibits the exchange between the cerebrospinal and interstitial fluids, impairing of solute clearance [1, 12, 22]. These mechanisms are plausible explanations for the development of cSVD. Second, factors that affect PI itself, such as age, high blood pressure, blood viscosity, and cardiac function, can also act as risk factors for cSVD [25].

Lastly, increased PI may be the result of increased downstream resistance due to the cSVD lesion itself, rather than the cause [13]. Indeed, our data and previous studies show a close relationship between PI and WMH/OLI, which are lesions of relatively large size. On contrary, CMB and EPVS did not show a clear relationship despite the pathological mechanism that explained their relationship with PI. However, as shown in Fig 1, there is a quantitative relationship between PI and EPVS tertiles. In addition, according to a previous study [12], the results of a multivariable analysis performed in patients with only mild cSVD burden showed that PI was clearly associated with the highest EPVS tertile (S2 Table). These results indicate that PI does not simply reflect the results of large cSVD lesions. Finally, both the causal and outcome relationships seem to be involved in the close association between PI and cSVD.

Unlike previous studies, we examined the relationship between PI and all kinds of cSVD subtypes. Because of the limitations of cross-sectional studies, it is difficult to prove the causal relationship between two with only our results. However, based on our results, we can receive impression that the increased arterial resistance may be a common pathological mechanism of cSVD, especially in WMH or OLI. Recently, studies have been conducted to reduce the arterial resistance using cilostazol or pravastatin in patients with cSVD or lacunar stroke [31, 32]. Therefore, as an extension of current study, it may result in interesting findings to design a prospective study to see if these medications 1) not only reduce the arterial resistance measured by PI in patients with lacunar stroke, 2) but also reduce the progression of WMH volume or the additional development of OLI lesions.

### Limitations

This study has several limitations. First, as a single-center retrospective study, there is a possibility of selection bias. Second, due to the limitation of the cross-sectional analysis, we were not able to prove causality. Even if we analyzed the relationship between PI and cSVD in each hemisphere and considered location information, several questions still need to be answered to determine whether this relationship is causal or consequential. Additional prospective cohort study is needed to identify the causal relationship. Third, this study did not directly measure arterial stiffness. If we include indicators that directly reflect the arterial stiffness, such as pulse wave velocity, and compared the results with PI, it would have helped to determine whether the close relationship between PI and cSVD was the cause or the result. Fourth, if we could measure the cerebral arterial pressure directly during systolic and diastolic phase, we could get a more intuitive impression to explain the mechanism between high PI and cSVD. Fifth, as mentioned earlier, it is possible that PI may have acted as a simple surrogate of previously known risk factors of cSVD. Finally, PI can be affected by various medical conditions [25]. Therefore, our results should be interpreted in view of these points.

## Conclusion

We demonstrated that ipsilateral PI is associated with cSVD in patients with lacunar stroke. PI clearly reflected the burden as well as the prevalence of cSVD, but it is not yet known whether the relationship between the two is causal or simply an indicator of the final lesion. Either way, however, it may be helpful to use PI in studies seeking to identify pathological mechanisms interconnecting arterial resistance and cSVD development.

## Supporting information

S1 Table Univariate linear regression analysis between PI and risk factors/radiological parameters. (DOCX)

S2 Table Univariate and multivariable logistic regression analyses between possible predictors and the 3^rd^ tertile enlarged perivascular space in patients with mild burden of cerebral small vessel diseases. (DOCX)

S1 Dataset (XLSX)
