# Supplementary material for: Distinct association between cerebral arterial pulsatility and subtypes of cerebral small vessel disease
Source: PLoS One. 2020 Jul 16;15(7):e0236049. doi: 10.1371/journal.pone.0236049 (PMC7365409; doi:10.1371/journal.pone.0236049)
Supplement: S1 Table — (DOCX) [file pone.0236049.s001.docx]

**S1 Table. Univariate linear regression analysis between PI and risk factors/radiological parameters**

|  | **Univariate** **analysis** | |
| --- | --- | --- |
|  | **B (95% CI)** | ***P* value** |
| Age | 0.008 (0.007 to 0.009) | < 0.001 |
| Sex, male | -0.018 (-0.056 to 0.020) | 0.355 |
| Hypertension | 0.002 (-0.035 to 0.038) | 0.932 |
| Diabetes | 0.046 (0.007 to 0.084) | 0.020 |
| Hyperlipidemia | -0.025 (-0.062 to 0.012) | 0.182 |
| Current smoking | -0.057 (-0.092 to -0.022) | 0.002 |
| Initial NIHSS^*^ | 0.014 (-0.012 to 0.041) | 0.282 |
| Use of antihypertensive | 0.023 (-0.012 to 0.059) | 0.196 |
| Use of lipid-lowering agents | -0.008 (-0.049 to 0.033) | 0.708 |
| DWI volume^*^ | 0.001 (-0.016 to 0.018) | 0.922 |
| WMH volume^*^ | 0.049 (0.038 to 0.061) | < 0.001 |
| Old lacunar infarct | 0.077 (0.042 to 0.113) | < 0.001 |
| Cerebral microbleed | 0.022 (-0.017 to 0.062) | 0.267 |
| EPVS number | 0.007 (0.004 to 0.010) | < 0.001 |

NIHSS = National Institutes of Health Stroke Scale, DWI = diffusion-weighted imaging, WMH = white matter hyperintensity, EPVS = enlarged perivascular space

^*^These variables were transformed into a square root scale.
