# Supplementary material for: Distinct association between cerebral arterial pulsatility and subtypes of cerebral small vessel disease
Source: PLoS One. 2020 Jul 16;15(7):e0236049. doi: 10.1371/journal.pone.0236049 (PMC7365409; doi:10.1371/journal.pone.0236049)
Supplement: S2 Table — (DOCX) [file pone.0236049.s002.docx]

**S2 Table. Univariate and multivariable logistic regression analyses between possible predictors and the 3^rd^ tertile enlarged perivascular space in patients with mild burden of cerebral small vessel diseases^*^**

|  | **Univariate** **analysis** | | **Multivariable analysis** | |
| --- | --- | --- | --- | --- |
|  | **OR (95% CI)** | ***P* value** | **aOR (95% CI)** | ***P* value** |
| Age | 1.07 [1.02-1.11] | 0.002 | 1.04 [0.99-1.09] | 0.098 |
| Sex, male | 0.50 [0.23-1.09] | 0.080 | … | … |
| Hypertension | 1.35 [0.62-2.94] | 0.458 | … | … |
| Diabetes | 0.78 [0.31-1.92] | 0.583 | … | … |
| Hyperlipidemia | 0.73 [0.33-1.61] | 0.435 | … | … |
| Current smoking | 0.82 [0.38-1.78] | 0.620 | … | … |
| Initial NIHSS^†^ | 2.35 [1.16-4.78] | 0.018 | 2.01 [0.97-4.16] | 0.062 |
| Use of antihypertensive | 1.06 [0.49-2.29] | 0.897 | … | … |
| Use of lipid-lowering agents | 1.48 [0.53-4.10] | 0.457 | … | … |
| DWI volume^†^ | 1.30 [0.97-1.73] | 0.081 | 1.35 [0.98-1.85] | 0.063 |
| Pulsatility index | 62.95 [6.03-656.75] | 0.001 | 18.09 [1.28-254.96] | 0.032 |

NIHSS = National Institutes of Health Stroke Scale, DWI = diffusion-weighted imaging

^*^Mild burden of cerebral small vessel disease was defined as having first tertile of WMH volume, and absence to single old lacunar infarct and cerebral microbleed.

^†^These variables were transformed into a square root scale.
